# Anxiety and curiosity in hierarchical models of neural emotion processing—A mini review

**DOI:** 10.3389/fnhum.2024.1384020

**Published:** 2024-06-19

**Authors:** Christin Hilmerich, Markus J. Hofmann, Benny B. Briesemeister

**Affiliations:** ^1^Department of Psychology, University of Wuppertal, Wuppertal, Germany; ^2^Department of Psychology, IU International University, Erfurt, Germany; ^3^Deloitte Neuroscience Institute, Deloitte Consulting GmbH, Berlin, Germany

**Keywords:** anxiety, curiosity, trait, ACC, NAcc, fMRI, emotion, emotion regulation

## Abstract

Traditionally, two fundamentally different theoretical approaches have been used in emotion research to model (human) emotions: discrete emotion theories and dimensional approaches. More recent neurophysiological models like the hierarchical emotion theory suggest that both should be integrated. The aim of this review is to provide neurocognitive evidence for this perspective with a particular focus on experimental studies manipulating anxiety and/or curiosity. We searched for evidence that the neuronal correlates of discrete and dimensional emotional systems are tightly connected. Our review suggests that the ACC (anterior cingulate cortex) responds to both, anxiety, and curiosity. While amygdala activation has been primarily observed for anxiety, at least the NAcc (nucleus accumbens) responds to both, anxiety and curiosity. When these two areas closely collaborate, as indicated by strong connectivity, this may indicate emotion regulation, particularly when the situation is not predictable.

## 1 Introduction

Emotions are an essential element in our daily lives. They influence our actions and shape us as people. Traditionally, two predominant, concepts describing emotions have been competing, that is dimensional theories such as Wundt (1922), assuming that the emotional experience can be described in terms of a limited number of bipolar dimensions on the one hand, and concepts that describe emotions as a way more diverse set of distinct functional units on the other — an idea that goes back to Darwin (1877). The two conceptions have traditionally been seen as opposing alternatives (Russell, 1980), but more recent work relying on neurophysiology emphasizes their complementing qualities (Briesemeister et al., 2014). Most prominent in this regard is the work by Panksepp (2011). Using direct electrical stimulation in animal models to achieve causal effects, Panksepp proposed that emotions are processed hierarchically on three distinct levels. The primary level deals with discrete emotions that arise from subcortical processes. At the secondary level, emotions are transformed into conditioned responses, thus making them adaptable to different environments and situational factors. Finally, tertiary emotions support the dimensional approach and serve the conscious evaluation of (high level) affective characteristics (Panksepp, 1989).

Panksepp’s hierarchical theory of emotions is heavily based on neurophysiology, providing a detailed documentation of the neuronal pathways involved in emotion processing. It does not, however, provide detailed information on the functional-psychological dimension of emotions, despite the fact that the primary emotional systems, namely SEEKING, LUST, FEAR, CARE, RAGE, PLAY and PANIC, are described as distinct functional entities. The why and how to elicit the respective emotions without electrical stimulation remains a challenge for psychological theorizing for human participants. Interestingly, there exists a second, less well-known theory of emotion, the so-called Zurich Model of Social Motivation (ZMSM, Bischof, 1975, 1993), which partially fills that gap. To the best of our knowledge, Panksepp and Bischof did not know of each other when initially postulating their respective theories, yet they show a remarkable overlap with regards to the emotions they consider primary. While Panksepp’s seven distinct, primary emotional systems are the result of electrostimulation studies, Bischof focuses on a functionalist structure equation model, arriving at six primary emotional states. Five of these states (curiosity, fear, dominance, submission and bonding) are quite equivalent or at least very similar to Panksepp’s emotional systems (SEEKING, FEAR, RAGE, PANIC, CARE). Interestingly, Bischof (1975) conceptualizes these emotions as functionally distinct ends on three continuous affective dimensions. Here we focus on the arousal dimension that can lead to curiosity or fear (Bischof, 1985). This depends on the question whether the “individual […] is more likely to explore a novel situation [rather] than to withdraw from it”—these individuals are called “enterprising” (Bischof, 1975, p. 809).

## 2 A prime example for emotion research: the case of anxiety

Anxiety is one of the few emotions that is considered in nearly every discrete emotion theory of emotion ever published. This makes anxiety an excellent example when comparing and explaining different theoretical approaches. From the ZMSM/hierarchical model perspective, anxiety is part of the FEAR (Panksepp denomination) or arousal system (Bischof’s denomination). Therefore, anxiety is embedded in a homeostatic system for responding to unknown and thus potentially threatening stimuli. According to Bischof, the opposite pole of this bipolar homeostatic system is not courage, as often assumed, but curiosity (Bischof, 1985). Both, anxiety, and curiosity are directly dependent on the amount of information that is available, relative to the amount of information that a person feels comfortable with — the latter is defined as the personality feature “enterprise” (Bischof, 1985). Enterprise refers to the amount of novel information that people strive for at any given moment, and even though it is expected to vary inter-personally, it is also considered to be fundamentally stable over time intra-personally and can therefore be considered a personality trait. Consequently, a high enterprise means that more information is sought, and such a person tends to exhibit highly curios behavior. Conversely, a low value means that any information exceeding the enterprise will cause anxiety (Bischof, 1985).

## 3 Current state of research

### 3.1 Anxiety

From a psychological perspective, one can distinguish between state and trait anxiety (Comte et al., 2015). The main difference between them lies in their temporal duration. While state anxiety represents an acute reaction to a subjectively threatening situation, anxiety as a trait exhibits a temporally persistent character that recurs throughout life and is thus considered a personality trait (Daviu et al., 2019). The link to Bischof seems apparent. State anxiety is characterized by very high arousal in each situation, i.e., the actual value of the ZMSMs security system is affected. Trait anxiety, on the other hand, is best described by a low enterprise within the ZMSMs security system, so that even every day and regularly repeated situations may trigger anxiety.

When trying to bridge the gap between Bischof’s psychometric approach and state-of-the-art neuroimaging studies, there are many experimental studies examining anxiety as a trait and anxiety as a state. They have identified a distributed network of brain areas involved in processing anxiety: Hypothalamus, amygdala, the cingulate cortex, the prefrontal cortex, and nuclei of the brainstem, in addition to the medial prefrontal cortex (mPFC), the locus coeruleus (LC) and reward processing areas such as the NAcc (Gross and Canteras, 2012; Felix-Ortiz et al., 2013; Janak and Tye, 2015; Daviu et al., 2019). Researchers like LeDoux and Pine (2016) have realized studies that investigated the amygdala, NAcc, prefrontal cortex, hypothalamus, and LC in emotional valence, stress, and anxiety, identifying the amygdala as the central structure from which reactions and actions of anxiety are being controlled. Jacobs et al. (2012) have also already investigated the fact that anxious people are constantly searching for anxiety-inducing stimuli. This connection between attention and anxiety is important.

Connections from the lateral to the central nucleus of the amygdala control anxiety responses, while connections from the LC to the basal nucleus (BA) and from there to the ventral striatum (NAcc) control the execution of actions such as escape and avoidance (Slocombe et al., 2012). Furthermore, Slocombe et al. (2012) investigated the extent to which the NAcc is activated during avoidance behavior, demonstrating that the extent of activation and deactivation of the NAcc during avoidance behavior is associated with individual levels of anxiety (Slocombe et al., 2012). Therefore, not only the amygdala, but also the NAcc play a key role in the development or maintenance of deviant avoidance behavior in anxiety.

### 3.2 Curiosity

Curiosity is the tendency to seek out new and challenging interactions. It can help people to engage with unfamiliar events for finding more information (Lydon-Staley et al., 2019)—as predicted by the ZMSM.

Gruber and Ranganath (2019) examined the extent to which curiosity as a cognitive state is related to exploration. Looking at functional imaging evidence, activity within the hippocampus, as well as the ACC were detected (Gruber and Ranganath, 2019). ACC activation is discussed to signal a cognitive conflict, arising from incongruent information (e.g., Botvinick et al., 2001), which in turn may elicit curiosity to find the information needed to resolve the conflict.

Furthermore, Huang et al. (2021) examined the relationship between anxiety, curiosity, interpersonal disengagement during the COVID-19 pandemic, and autistic tendency. They found a positive correlation between state anxiety and epistemic curiosity. In contrast, anxiety correlates negatively with interest-type curiosity, which means obtaining information that is expected to stimulate positive feelings interest. In contrast, deprivation-type curiosity, which means obtaining information to reduce undesirable states of informational deprivation, correlates positively with anxiety (Huang et al., 2021).

Given the theoretical assumptions of Bischof and Panksepp, as well as the largely separated literature on curiosity and anxiety, the present study aimed at searching published evidence for a functional neuro-anatomical network that comprises both emotions.

## 4 Selective review methods

To investigate a link between anxiety and curiosity on a neurophysiological level, even if only one of the emotions has been manipulated, we included these studies in our systematic literature search (Tranfield et al., 2003).

In a first step, the following keywords were used to find relevant publications: anxiety, curiosity, trait, and fMRI. After initial review of the limited number of found studies, a second search was conducted including the most common brain regions found in the first step. In the second step, we searched for the any combination of the keywords anxiety, curiosity, fMRI, ACC, emotion, emotion regulation and NAcc.

Publications were deemed relevant if they fulfilled the following criteria: anxiety as a trait and not as a disease and the application of neuroimaging to identify the respective neural networks. We also excluded animal studies, because neither Panksepp’s tertiary-cortical nor Bischof (1985) personality system can be investigated in these subjects. No book publications or anthologies were considered, nor any industry reports, conference reports, or articles in non-scientific journals. Altogether, 78 studies were identified as being potentially relevant. We also focused on publications within the last 10 years to only consider state-of-the-art research.

From April 2023 to July 2023, the databases PubMed, Science Direct and Frontiers were screened for articles including the combinations of the previously identified terms in the title, keyword, or abstract. After further thorough analysis of the selected 78 articles, another 67 articles were eliminated as inappropriate in content, because they reported no original experimental results including meta-analyses, which left us with 11 articles.

## 5 Results

Focusing on anxiety and curiosity Gruber and Ranganath (2019) investigated the connection of hippocampus and ACC with curiosity-based exploration as well as fear-based behavioral inhibition, using the PACE-framework (prediction, appraisal, curiosity, and exploration). Here, effects of curiosity and memory can be understood as emerging from a cycle that involves prediction errors, Appraisal, Curiosity, and Exploration. A pace cycle is completed once uncertainty is removed and curiosity is satisfied by closing an information gap. They found a connection of the hippocampus with the ACC in curiosity-based exploration and in fear-based behavioral inhibition.

Using a lottery task to manipulate curiosity, Van Lieshout et al. (2018) showed that curiosity could explain risk seeking behavior even if the situation is not predictable and thus potentially anxiety-inducing. Two potential sources of curiosity were manipulated independently: outcome uncertainty and expected value. The results show that participants are more curious in uncertain outcomes and when the expected value is low.

Van Lieshout et al. (2021a) further added insights by investigating whether curiosity regarding uncertain outcomes is modulated by the valence of the information, again using a lottery task. Overall curiosity for wins was higher than for losses and curiosity increased with increasing outcome uncertainty for both wins and losses, demonstrating that the effect is independent of the outcome valence.

Finally, Van Lieshout et al. (2021b) manipulated the uncertainty of gains or losses. They found that the test subjects were more satisfied and curious with winning lotteries than with losing lotteries, especially if the uncertainty of the outcome was low and the expected prize was high.

Focusing on anxiety rather than both emotions, Eden et al. (2015) examined the differences of individuals with anxious traits and less anxious traits to determine which areas of the cortex are involved in emotion regulation. They collected diffusion-weighted magnetic resonance images and applied probabilistic tractography. Results showed anatomic connectivity between the amygdala and the ACC when participants showed a skill in emotion regulation.

Comte et al. (2015) examined the relationship between trait anxiety and functional connectivity of the ACC with the lateral prefrontal cortex, using an emotional conflict task. The central part of the image displayed photographs of faces expressing positive emotion or negative emotion. The peripheral part, on which the face images were superimposed, represented scenes with a pleasant or unpleasant emotional content. Results show that higher levels of anxiety were associated with greater task-related activation in ACC, but with reduced functional connectivity between ACC and lateral prefrontal cortex.

Focusing on curiosity rather than anxiety, Wei et al. (2022) were interested in the structural brain circuitry related to novelty seeking. Using an MRI scan and the Revised Temperament and Character Inventory (TCI), they examined the structural connections between the cerebellum and the cerebral cortex, the thalamus and the basal ganglia, finding strong connections between these structures. A score was also determined for all personality traits according to Cloninger’s psychobiological personality model.

Lau et al. (2020) asked the question: Which brain regions predict curiosity behavior in the presence of danger? Participants watched videos of food (control) or magic tricks and were then asked to rate how much they would like to consume the food or see the resolution of the trick. They were subsequently given the option to participate in a lottery or not. If they participated and won in the lottery, the magic trick was resolved; if they lost, they were threatened with electrocution. The probability of winning was manipulated and communicated in each round trial. The study found that people are willing to take risks and even endure electric shocks to satisfy their curiosity, even if the knowledge gained has no obvious benefit.

Gruber et al. (2014) used fMRI to investigate how curiosity influences memory. The results of functional magnetic resonance imaging showed that activity in the midbrain and in the NAcc was increased in states of high curiosity during learning.

Eschmann et al. (2023a) examined whether curiosity predicts different characteristics of information seeking in real life and whether functional connectivity within the mesolimbic dopaminergic circuit is associated with information seeking outside the laboratory. This study was conducted using curiosity and anxiety questionnaires and a 10-min resting-state functional magnetic resonance imaging session. Participants repeated this in a follow-up survey during the COVID-19 pandemic. Results show that curiosity is associated with the mesolimbic dopaminergic functional network, supporting information-seeking behavior in real life. High ratings of curiosity showed increased activity in the ventral tegmental area (VTA) and the NAcc.

Eschmann et al. (2023b) also investigated whether individual differences in functional connectivity, as measured by resting-state fMRI, determine the extent to which individuals benefit from the memory-enhancing effects of curiosity and information prediction errors (IPEs–the discrepancy between information and expected expectations). The results show that individual differences in bilateral functional connectivity between ACC and left hippocampus determine the degree to which individuals benefit from the memory-enhancing effects of curiosity, but not from the memory enhancements caused by information prediction errors.

The results are summarized in Table 1.

**TABLE 1:** Results.

References	Emotion	Description	Results
Gruber and Ranganath, 2019	Anxiety and curiosity	PACE-framework – Connection of hippocampus and ACC with curiosity-based exploration – Fear-based behavioral inhibition	– Connection of hippocampus with the ACC in curiosity-based exploration as well as within fear-based behavioral inhibition
Van Lieshout et al., 2018	Anxiety and curiosity	Lottery task – Risk-taking behavior due to curiosity when the situation is unpredictable	– More curious when the information gaps are larger corresponding to uncertainty
Van Lieshout et al., 2021a	Anxiety and curiosity	Lottery task – Curiosity for uncertain results is influenced by the value of the information	– Curiosity for wins was higher than for losses – Curiosity increased with increasing outcome uncertainty for both wins and losses
Van Lieshout et al., 2021b	Anxiety and curiosity	Lottery task – Manipulation of the uncertainly of gains or losses	– More satisfied and curious with winning lotteries than with losing lotteries
Eden et al., 2015	Anxiety	Magnetic resonance images and applied probabilistic tractography – Differences of individuals with anxious trails and less anxious traits – Determine which areas of the cortex are involved in emotion regulation	– Anatomic connectivity between the amygdala and the ACC when participants showed a skill in emotion regulation
Comte et al., 2015	Anxiety	Emotional conflict task – Relationship between trail anxiety and functional connectivity of the ACC with the lateral prefrontal cortex	– Higher levels of anxiety were associated with greater task-related activation in ACC, but with reduced functional connectivity between ACC and lateral prefrontal cortex
Wei et al., 2022	Curiosity	MRI scan and (TCI) – Examined the structural connections between the cerebellum and the cerebral cortex, the thalamus and the basal ganglia	– Strong connections between these structures. – A score was also determined for all personality traits according to Cloninger’s psychobiological personality model
Lau et al., 2020	Curiosity	Observation and survey – Which brain regions predict curiosity behavior in the presence of danger – Rate how much they would like to consume the food or see the resolution of the trick	– People are willing to take risks and even endure electric shocks to satisfy their curiosity, even if the knowledge gained has no obvious benefit
Gruber et al., 2014	Curiosity	fMRI – Investigate how curiosity influences memory	– Activity in the midbrain and in the NAcc was increased in states of high curiosity during learning
Eschmann et al., 2023a	Curiosity	fMRI – Predicting different characteristics of information seeking by curiosity	– Curiosity is associated with the mesolimbic dopaminergic functional network, supporting information seeking behavior in real life – High ratings of curiosity showed increased activity in the ventral tegmental area (VTA) and the NAcc
Eschmann et al., 2023b	Curiosity	fMRI – Individual differences in functional connectivity, determine the extent to which curiosity and information prediction errors arise	– Individual differences in bilateral functional connectivity between ACC and left hippocampus – Not from the memory enhancements caused by information prediction errors.

## 6 Discussion

The current review aimed at searching for published evidence for a functional neuro-anatomical network that comprises both emotions, anxiety and curiosity, as opposing poles of a single emotional/motivational dimension as has been suggested by Bischof (1975, 1993) theoretical framework. The review of the 11 studies, which were all experimental in design and manipulated either anxiety as a state, curiosity or both, revealed the following three important structures: the amygdala as a central hub in anxiety processing (Comte et al., 2015; Eden et al., 2015), the NAcc as a central structure within curiosity processing (Gruber et al., 2014; Eschmann et al., 2023a), and the ACC as a central hub in both, curiosity processing and anxiety (Gruber and Ranganath, 2019).

The most crucial brain areas involved in anxiety as a state are the hypothalamus, amygdala, cingulate cortex, prefrontal cortex, brain stem nuclei, LC, and NAcc, with the amygdala being referenced as the core area associated with anxiety. But not only the amygdala is essential–there seems to be strong connectivity between amygdala and ACC in emotion regulation when the situation is not predictable, as Eden et al. (2015) show. This is of note, because the ACC is involved in both emotion processing and cognitive control, and the connection to the amygdala may serve to evaluate emotional stimuli and trigger appropriate behavioral responses.

The ACC also was found to be active as part of a curiosity network, as a way to make a decision and to regulate emotions. Yagi et al. (2023) showed that curiosity is an explanation for risk seeking behavior despite anxiety or uncertainty, especially when the situation is not predictable. People are more likely to open a presented box, when the outcome is uncertain, and expected to be negative than when the outcome is certain and neutral or certain and negative. Furthermore, the NAcc is often associated with approach motivation, as would be expected from curiosity (Gruber et al., 2014; Eschmann et al., 2023a). The results of Lau et al. (2020) show that the NAcc predicts curiosity behavior even in the presence of danger.

Taken together, these studies can be interpreted as initial support for the hypothesis that anxiety and curiosity are two distinct poles, with clearly distinctive functional meaning (approach vs. withdrawal), yet part of the same affective dimension (e.g., the arousal dimension as suggested by Bischof, 1985). Both, studies investigating anxiety (Comte et al., 2015; Eden et al., 2015) and studies investigating curiosity (Eschmann et al., 2023a), show activity changes within the ACC, a structure known to also be associated with the processing of prediction errors (Eschmann et al., 2023b) and conflict monitoring (Botvinick et al., 2001). The ACC is assumed to monitor and evaluate actions and their consequences (Botvinick et al., 2001), which according to the conflict monitoring hypothesis requires a comparison of expectations and actual outcomes. Thus, our overall understanding of ACC functions is well in line with the assumptions of the ZMSM. In the model from Bischof, the comparison of expected and actual values of the security system is the formal basis for an emotional response. Depending on the outcome of this comparison and on the expectancy level of the participant, a mismatch might occur, which may lead to curiosity, when stimulus arousal is lower than (delight in) enterprise. Anxiety is induced, in contrast, when stimulus arousal is higher than enterprise. Anxiety refers to the response to unknown and thus potentially threatening stimuli, which are processed within the amygdala. This leads to withdrawal motivation. Curiosity also refers to the response to unknown situations, but in contrast leads to approach motivations toward those to potentially threatening stimuli (Gruber and Ranganath, 2019). This is initial evidence for a complementary role of anxiety and curiosity, acting as two functionally distinct poles on an emotional continuum or, in terms of Bischof, both emotions form a bipolar homeostatic system (Bischof, 1985). We suggest that this perspective reconciles the traditionally opposing views of functionally discrete emotions and theories assuming a limited number of affective dimensions.

**FIGURE 1:**
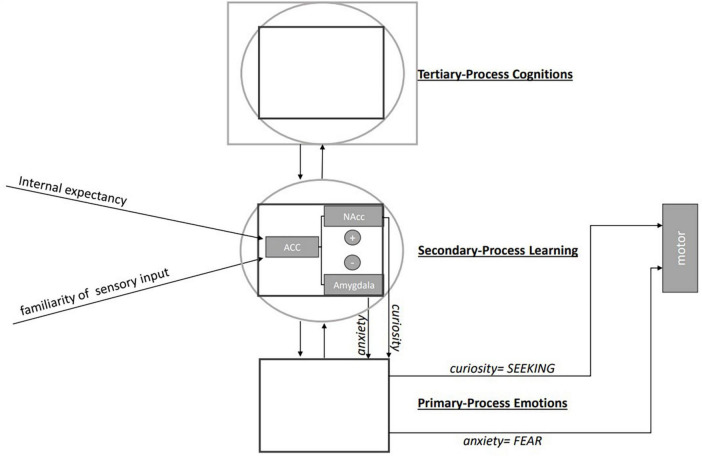
Hierarchical processing of emotions: From stimulus evaluation to affectively controlled motor response.

Though the current literature provides no direct test of the ZMSM, it supports the assumptions of an initial comparison of subjective expectancies with objective external information within the ACC. This leads to stimulus evaluation and in in turn triggers an affectively driven motor response to either approach (curiosity, NAcc) or withdraw (anxiety, amygdala) from the situation. The connections between the relevant structures, namely ACC, NAcc and amygdala are well documented, but the hierarchical processing flow suggested by the ZMSM and also Panksepp (2011) hierarchical model of emotion (Figure 1) have not yet been well elaborated. Here we aimed to provide such a perspective to pave the way for future experimental research. The critical assumption here is that the same stimulus can lead to anxiety or curiosity, solely depending on the subjective expectations of the perceiving individual. This suggests a bipolar homeostatic system (Bischof, 1985) at the transition from secondary to primary process level emotions (Panksepp, 2011).

## Author contributions

CH: Conceptualization, Writing – original draft, Writing – review & editing. MH: Supervision, Writing – review & editing. BB: Supervision, Writing – review & editing.
